# Development of a Novel Typing Scheme Based on the Genetic Diversity of Heme/Hemin Uptake System Hmu in *Klebsiella pneumoniae* Species Complex

**DOI:** 10.1128/spectrum.01062-22

**Published:** 2023-02-14

**Authors:** Peng Lan, Ye Lu, Rushuang Yan, Lei Fang, Dongdong Zhao, Yan Jiang, Yunsong Yu, Xiaoxing Du, Jiancang Zhou

**Affiliations:** a Department of Critical Care Medicine, Sir Run Run Shaw Hospital, Zhejiang University School of Medicine, Hangzhou, China; b Key Laboratory of Microbial Technology and Bioinformatics of Zhejiang Province, Hangzhou, China; c Department of Infectious Diseases, Sir Run Run Shaw Hospital, Zhejiang University School of Medicine, Hangzhou, China; The Pennsylvania State University

**Keywords:** *Klebsiella pneumoniae*, hemin, genetic diversity, iron, Hmu

## Abstract

Iron is essential for the survival and reproduction of Klebsiella pneumoniae. Although K. pneumoniae employs multiple types of siderophores to scavenge iron during infections, the majority of host iron is retained within erythrocytes and carried by hemoglobin that is inaccessible to siderophores. HmuRSTUV is a bacterial hemin/hemoprotein uptake system. However, the genetic background and function of HmuRSTUV in K. pneumoniae remain unknown. We collected 2,242 K. pneumoniae genomes, of which 2,218 (98.9%) had complete *hmuRSTUV* loci. Based on the 2,218 complete *hmuRSTUV* sequences, we established a novel typing scheme of K. pneumoniae named hmST, and 446 nonrepetitive hmSTs were identified. In hypervirulent lineages, hmST was diversely distributed and hmST1 mainly existed in ST23 strains. In contrast, hmST was less diversely distributed among multidrug-resistant strains. hmST demonstrated greater genetic diversity in hypervirulent lineages and community-acquired and bloodstream-sourced strains. *In vitro* and *in vivo* experiments revealed that an intact *hmuRSTUV* was essential for hemin uptake, playing an important role in bloodstream infections. This study established a novel typing scheme of hmST based on *hmuRSTUV* providing new insights into identifying and monitoring the emergence of novel virulence evolution in K. pneumoniae.

**IMPORTANCE** Siderophore is a group of low molecular weight compounds with high affinity for ferric iron, which could facilitate bacterial iron consumption. Similarly, hemin/heme scavenged by the hemin uptake system HmuRSTUV usually act as another critical iron source for K. pneumoniae. This study proved that Hmu system significantly promoted the growth of K. pneumoniae in the presence of hemin and played an important role in bloodstream infections. A novel typing scheme named hmST was established, and the genetic diversity of *hmuRSTUV* loci was analyzed based on a large number of genomes. This study provides new insights into identifying and monitoring the emergence of novel virulence evolution in K. pneumoniae.

## INTRODUCTION

Klebsiella pneumoniae is recognized as an urgent problem of public health, where the emergence and dissemination of multidrug-resistant (MDR) and hypervirulent lineages has contributed to the establishment of health care-associated infection outbreaks. Most hypervirulent *K. pneumoniae* (hvKP) strains belong to the K1/K2 serotype, which frequently lead to clinically invasive syndromes, including liver abscess, central nervous system infection, necrotizing fasciitis, and endophthalmitis (1). K1 strains generally belong to the ST23 lineage, whereas K2 is found in more diverse clonal backgrounds (such as ST65, ST86, ST380, etc.) (2–4). In contrast, multidrug-resistant (MDR) K. pneumoniae strains cover distinct lineages, including ST11, ST15, and ST258 (5, 6). Despite the distinct genetic background, both hypervirulent and MDR lineages are heavily dependent on iron to sustain growth during infection (7–9).

Generally, ferric iron has a solubility of only 10^−17^ M at pH 7, while bacteria require iron at around 10^−7^ to 10^−5^ M to thrive (10). Iron is much less accessible in host (human), because it is frequently bound with proteins, such as ferritin, transferrin, and myoglobin (11). To satisfy the metabolic requirement, K. pneumoniae have evolved multiple strategies to steal iron from the environment, especially from the host during infections (7). Siderophore is one of the most important iron uptake systems for K. pneumoniae used to sequester iron. Siderophores are low-molecular-weight compounds with a high affinity for Fe^3+^ (12). K. pneumoniae can produce four types of siderophore, including enterobactin (Ent), salmochelin (Iro), yersiniabactin (Ybt), and aerobactin (Iuc) (13). These siderophores, especially aerobactin, significantly enhance the virulence of K. pneumoniae (14, 15). Consequently, the ability to acquire iron has served as an essential marker for virulence (14, 16).

**FIG 1:**
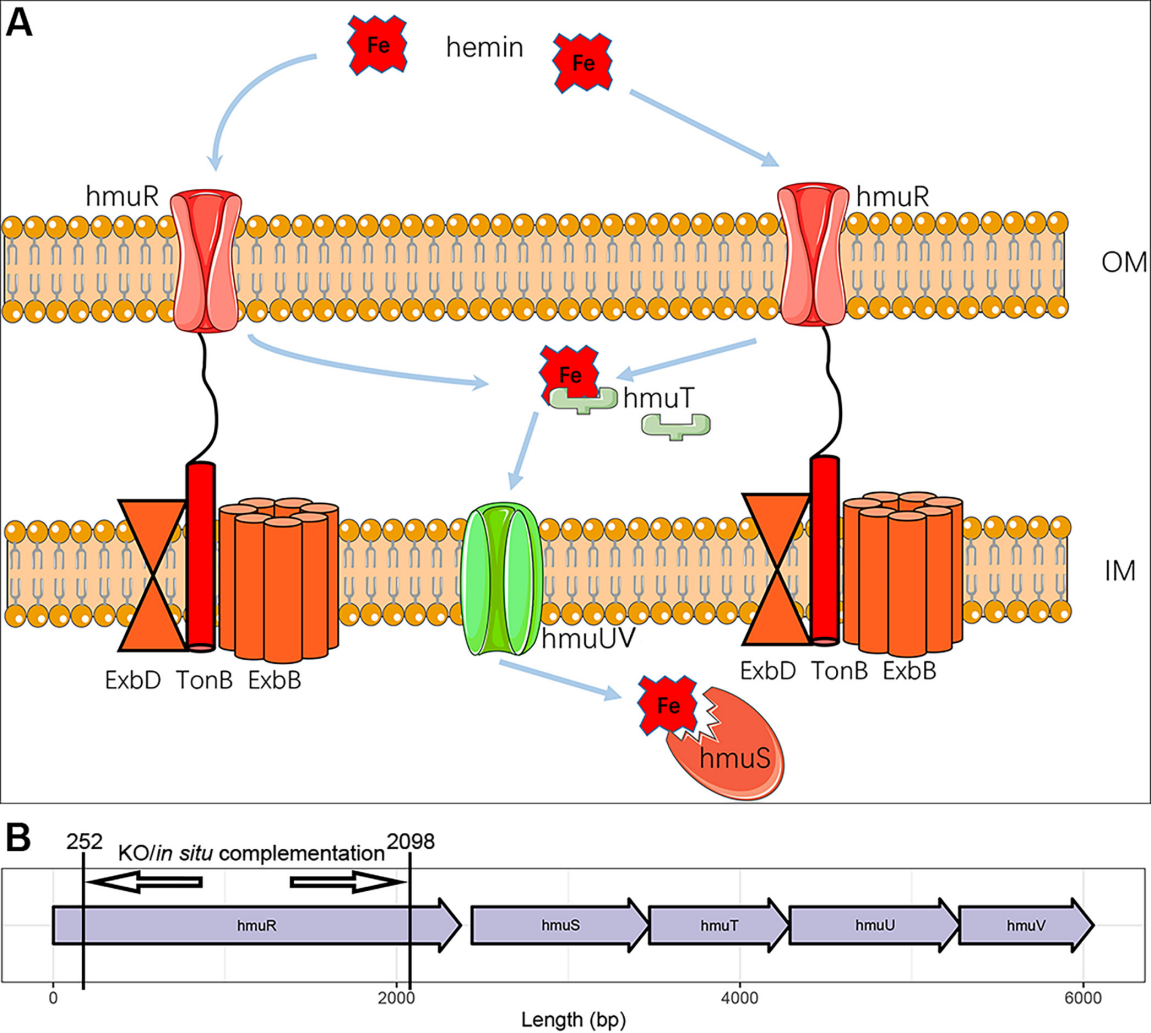
Composition of hemin uptake system in K. pneumoniae. (A) Model of the Hmu system contributing to hemin uptake in K. pneumoniae. (B) *hmuRSTUV* locus in K. pneumoniae. OM, outer membrane; IM, inner membrane.

Nevertheless, the majority of the host iron is located within erythrocyte hemoglobin (17), which comprises four subunits, each containing an iron atom bound to a heme group. This is a great “iron pool” that siderophores could not access. The mature erythrocyte remains in circulation for about 120 days until being engulfed and digested by phagocytes mainly in the liver, bone marrow, and spleen (17). Heme is then released from the phagolysosomes and degraded by the heme oxygenase-1, freeing iron. To prevent the toxicity caused by cell-free hemoglobin (CFH), haptoglobin removes CFH from plasma by forming high-molecular-weight haptoglobin-hemoglobin complexes (17, 18). Once the hemoglobin binding capacity of haptoglobin is exceeded, heme can be released into the blood directly from free hemoglobin (19). Hemin is the oxidized version of heme. K. pneumoniae can scavenge hemin/heme as an iron source via the HmuRSTUV hemin uptake system (7). Like siderophore receptors, HmuR is a TonB-dependent outer membrane receptor that is required for the utilization of heme (Fig. 1). HmuTUV constitute an inner membrane ABC transporter involving in the transportation of hemin into the cytoplasm, while HmuS is essential for the degradation of hemin and iron releasing (20). The biological function of Hmu system has been well characterized in *Yersinia* spp. (20, 21) but was far less explored in K. pneumoniae.

Molecular typing is critical for distinguishing bacterial strains and thus provides a good strategy for surveillance, outbreak investigation, and phylogenetic analyses. Traditional typing methods, including plasmid analysis, genome enzyme restriction, and pulsed-field gel electrophoresis, have been used for a long time for typing and clonal assignment (22). These methods require agarose gel electrophoresis to separate enzyme-restricted plasmid or genome DNA fragments. Multilocus sequence typing (MLST) based on the alleles of seven housekeeping genes (*gapA*, *infB*, *mdh*, *pgi*, *phoE*, *rpoB*, and *tonB*) is now widely performed in K. pneumoniae and serves as the standardized scheme used for parallel comparisons of strains from different sources. With the development of high-throughput sequencing techniques, large amounts of data generated by genome sequencing can be obtained in a short time at low cost. Core genome MLST (cgMLST) has been widely used to study the molecular epidemiological characteristics of K. pneumoniae owing to its higher resolution (23). However, these typing strategies are based on global bacterial genomes rather than specific aspects. To gain a deeper understanding of the genetic diversity of siderophores, novel typing schemes (including SmST, AbST, and YbST) based on siderophore-encoding loci have been developed and updated (https://bigsdb.pasteur.fr/klebsiella/) (24, 25). These novel typing methods provide an important resource to identify and monitor the emergence of undiscovered virulence evolution in K. pneumoniae. Considering that hemin is one of the critical sources of iron, we hypothesized that Hmu system might be involved in the virulence of K. pneumoniae. In this study, we aimed to (i) research the genetic diversity of *hmu* loci in K. pneumoniae and accordingly establish a novel typing scheme capable of tracking and tracing the virulence evolution and to (ii) demonstrate the role of Hmu system in bloodstream infections.

## RESULTS

### Establishment of hmST based on *hmuRSTUV* locus.

The *hmu* locus is located on the chromosome and is required for the hemin uptake in K. pneumoniae (Fig. 1). The genomes examined in this study included 1,970 genomes from Pathogenwatch (https://pathogen.watch/) and 272 genomes from our previous study, which covered 20 tertiary hospitals in China (23). All public collections of genomes available at Pathogenwatch were included in the study. The 2,242 strains of the K. pneumoniae species complex were from 34 countries. Most of these were K. pneumoniae (2,212/2,242, 98.7%) strains, while *K. variicola* and *K. quasipneumoniae* accounted for only 0.7% (16/2,242) and 0.6% (14/2,242) of the genomes, respectively. Among the 2,242 K. pneumoniae species complex genomes, 2,218 (98.9%) had intact *hmuRSTUV* sequences, and 5 lacked *hmuRSTUV*. Among the remaining 19 strains, *hmuR* was absent in five strains, *hmuS* was absent in three, *hmuT* was absent in five, *hmuU* in five, and *hmuV* was absent in one. Based on the 2,218 complete *hmuRSTUV* allelic profiles, we established a novel typing scheme named hmST. Each observed combination of alleles was assigned a unique hmST. Among the 2,218 genomes, 446 nonrepetitive hmSTs were identified (see Table S1 in the supplemental material). Complete allelic profiles of *hmuRSTUV* sequences were shown in Table S1.

### *hmuRSTUV* locus diversity in *K. pneumoniae*.

**FIG 2:**
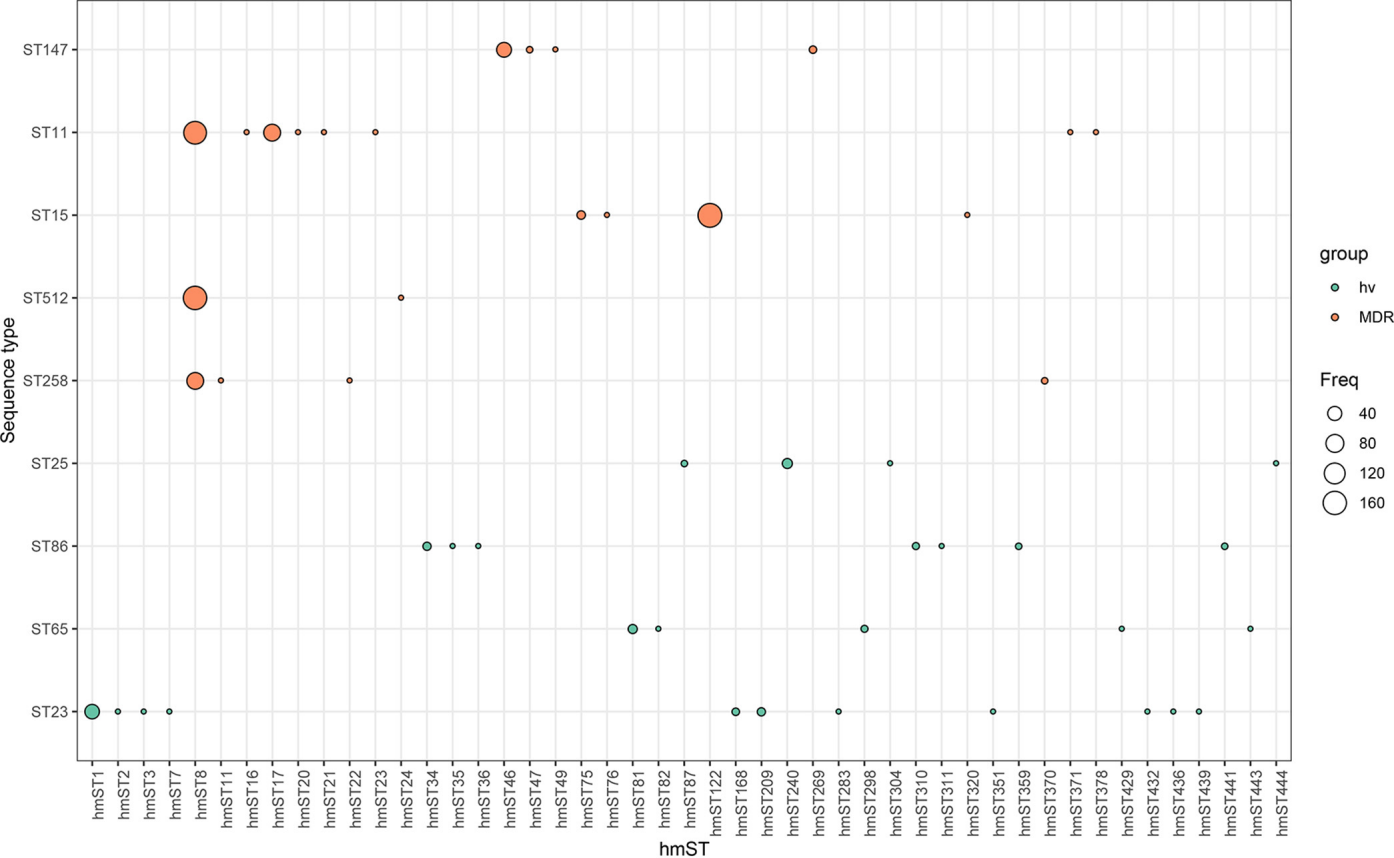
Frequency distribution of hmSTs in hypervirulent and MDR lineages. Hypervirulent lineages include ST23, ST65, ST86, and ST25, while MDR lineages include ST11, ST258, ST512, ST147, and ST15. The figure was generated by using R software (version 3.3.3). hv, hypervirulent; MDR, multidrug resistant.

**FIG 3:**
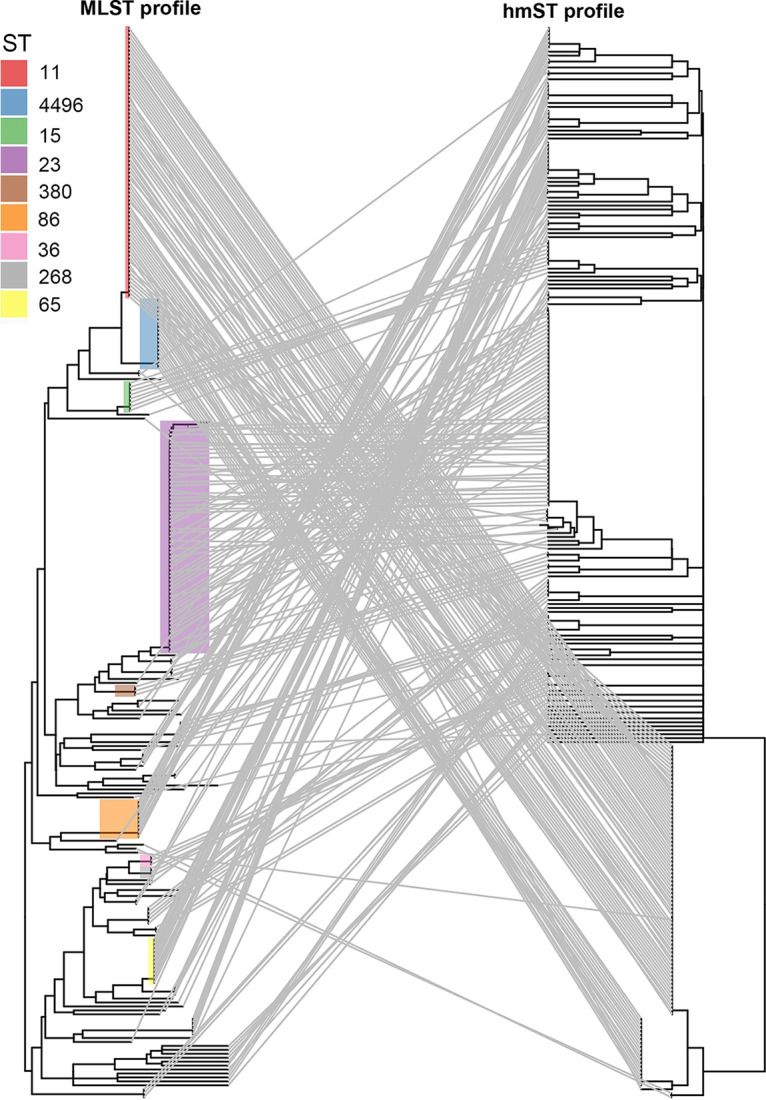
Phylogenies inferred from MLST (left) and hmST (right). Matching strains were connected using gray auxiliary lines. Strains were colored according to main sequence type based on seven-gene MLST. The figure was generated by using the ggtree package with R software.

To explore the association between hmST and common lineages, we compared the distribution of hmSTs among the most common hypervirulent (including ST23, ST86, ST65, and ST25) and MDR (including ST11, ST258, ST512, ST15, and ST147) lineages. As shown in Fig. 2, hmST8 only existed in ST11, ST258, and ST512 MDR strains, all of which belong to CG258. hmST122 and hmST46 accounted for the majority of the hmSTs in ST15 and ST147 lineages, respectively. In contrast, hmST was diversely distributed among hypervirulent lineages. For example, though hmST1 was only observed in hypervirulent ST23 strains, multiple other hmSTs, including hmST2, hmST3, hmST7, hmST168, hmST209, hmST283, hmST351, hmST432, hmST436, and hmST439, were also found in ST23 strains. Interestingly, hypervirulent and MDR lineages had no shared hmST. Genomes from our previous work were selected to build phylogenetic trees (23). As shown in Fig. 3, the phylogenetic tree of the MDR lineages ST11 and ST4496 based on MLST reflected nearly the complete phylogeny of the *hmuRSTUV* allelic profile. For hypervirulent ST23, the nonconcordance of the phylogeny of hmST and MLST phylogeny were observed (ST23 data are presented separately in Fig. S1), indicating that hmST might possess higher discriminatory efficacy in classifying hypervirulent lineages.

**FIG 4:**
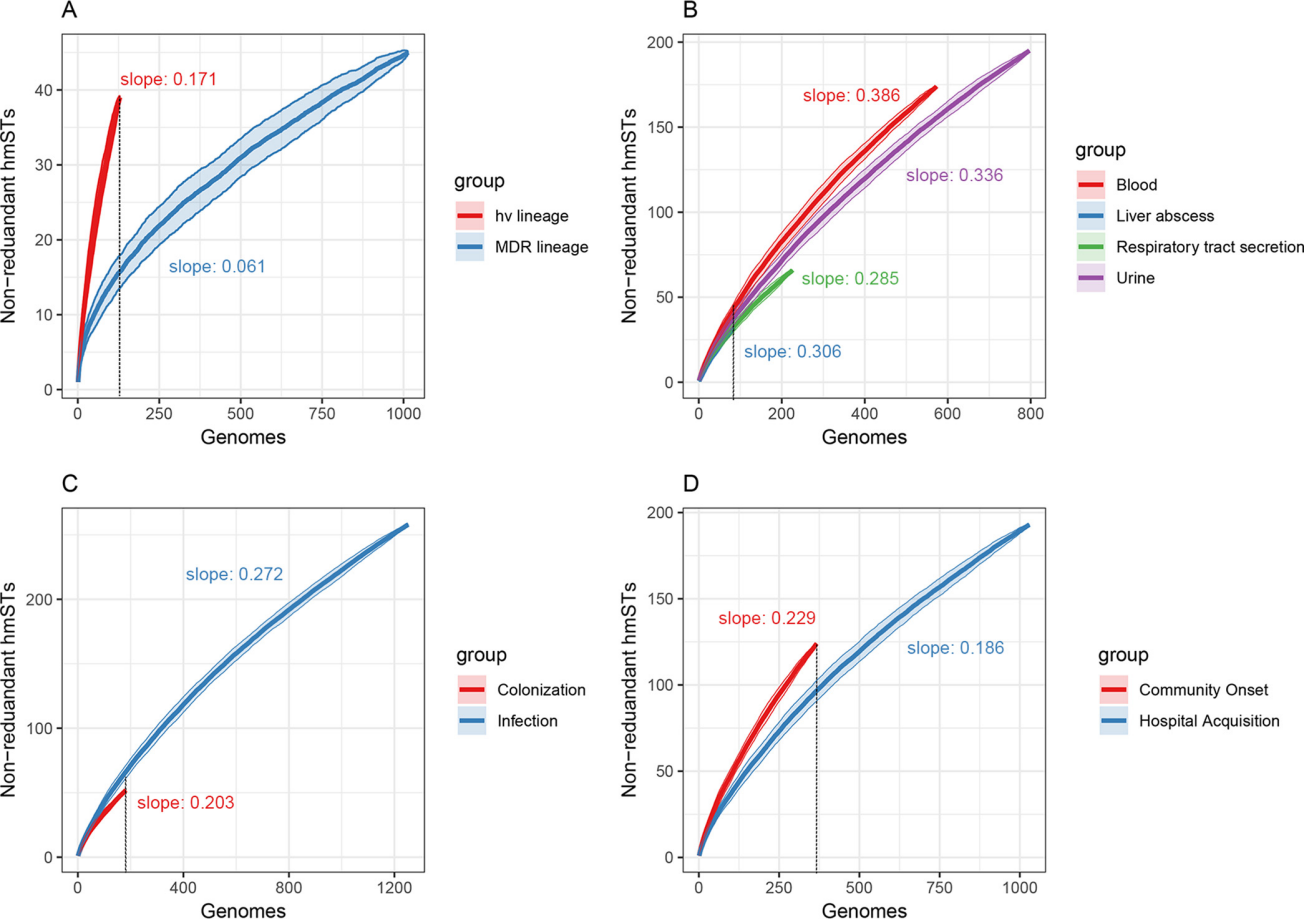
(A to D) Accumulation curves of hmST according to different lineages (A), sources (B), infection statuses (C), and infection types (D). Curves indicated the accumulation of distinct hmSTs (means ± the standard deviations) with increasing genome sample size in different genome sets. The slope for each curve was indicated in the figure. The figure was generated by using vegan and the ggplot2 package with R software. hv, hypervirulent; MDR, multidrug resistant.

We employed accumulation curves to assess the diversity of hmSTs among different lineages (hypervirulent and MDR lineages), sources (bloodstream, liver abscess, respiratory tract, and urinary tract), infection statuses (colonization and infection), and infection types (community onset and hospital acquired). As sampling frequency increased, more hmSTs were observed, resulting in the ascending accumulation curves. The slopes of the accumulation curves reflected the diversity of hmSTs. As shown in Fig. 4A, the accumulation curve for hypervirulent lineages was far steeper than that for MDR lineages, indicating that hmSTs were more diverse among hypervirulent lineages than those from MDR lineages. Similarly, hmSTs showed more diversity in bloodstream-sourced, infection-isolated, and community-onset strains compared to those from other infection sites (i.e., liver abscess, urinary tract, and respiratory tract), colonization and hospital-acquired strains (Fig. 4B to D).

### Role of Hmu in hemin uptake.

**FIG 5:**
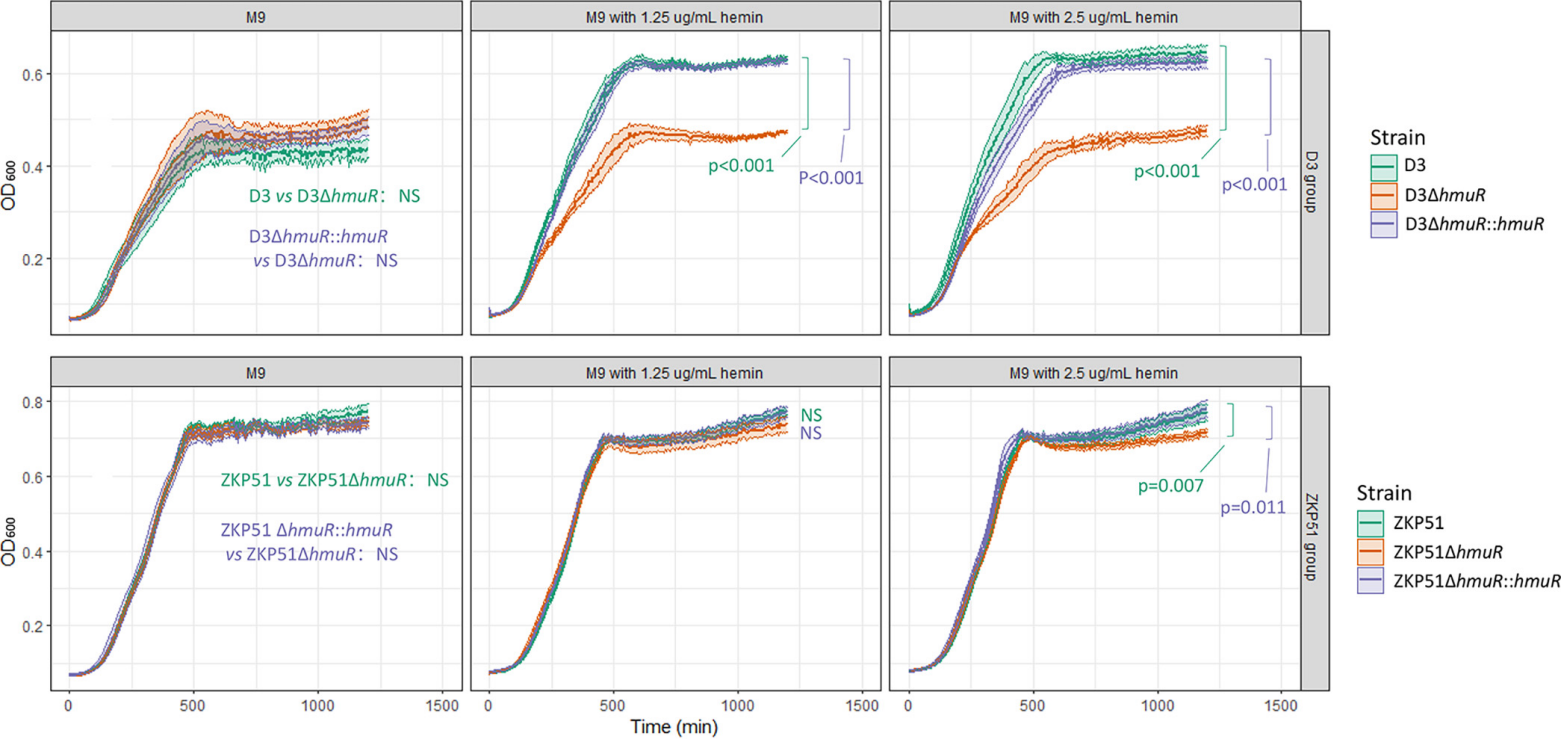
Growth curves of strain D3, ZKP51, and their mutants in M9 standard medium with or without hemin supplement. The OD_600_ values over time were presented. Values at each time point were depicted as means with confidence intervals (± the standard deviations). The growth rates of the mutants were compared to wild and complemented strains by using a Student *t* test.

Although the function of HmuRSTUV in *Yersinia* spp. has been well elucidated, research on the Hmu system in K. pneumoniae is extremely limited. It was found that the Hmu system was expressed at greater levels in *entS* (encoding an enterobactin exporter) mutant CRKP strains, compared to strains with intact *entS* (8). However, the role of the Hmu system in K. pneumoniae was not determined. To explore the role of Hmu in hemin uptake, we constructed *hmuR* knockouts and *hmuR* complementations in strains D3 and ZKP51 to compare their growth under conditions where hemin served as the unique iron source. Clinical isolates D3 and ZKP51 represented hypervirulent and MDR lineages, respectively. Knockout and complementation were performed by using a CRISPR-Cas9-mediated genome-editing method. In M9 standard medium without any supplement, all of the strains showed no growth difference (Fig. 5). However, when M9 medium was supplemented with bovine hemin, D3Δ*hmuR* and ZKP51Δ*hmuR* strains grew more slowly during exponential phase and then reached a lower plateau compared to their corresponding wild types. The complemented mutants could restore the growth superiority (Fig. 5). However, the growth discrepancy between *hmuR* mutants and wild-type strains (as well as complemented mutants) became larger as the hemin supplement increased from 1.25 to 2.5 μg/mL. These results suggested that the Hmu system was essential for K. pneumoniae to thrive in the presence of hemin.

### Role of Hmu in bloodstream infection.

**FIG 6:**
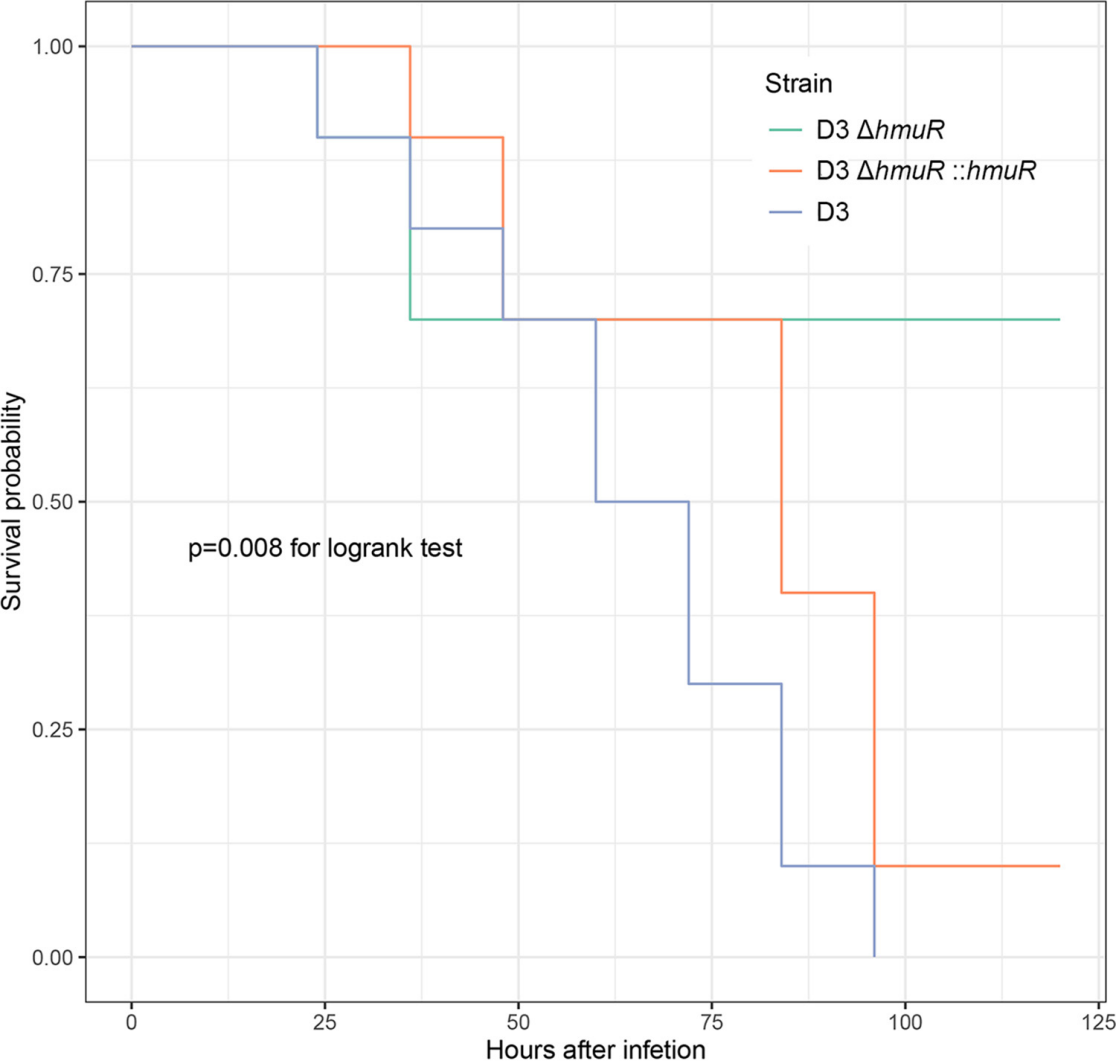
Survival curves of a mouse bloodstream infection model challenged by D3, D3Δ*hmuR*, and D3Δ*hmuR*::*hmuR* strains.

Since Hmu was essential in the hemin uptake process, we speculated that intact HmuRSTUV played an important role in bloodstream infections. A mouse lethality assay was performed using a bloodstream infection model for D3, D3Δ*hmuR*, and D3Δ*hmuR*::*hmuR* strains. As shown in Fig. 6, mice challenged with the D3Δ*hmuR* strain had lower risks of death than those challenged with the D3 and D3Δ*hmuR*::*hmuR* strains (*P* = 0.008 [log-rank test]). Although D3 and D3Δ*hmuR*::*hmuR* strains exhibited comparable virulence (hazard ratio, 0.529; 95% confidence interval [CI] = 0.209 to 1.340; *P* = 0.179, with strain D3 as a reference), the D3Δ*hmuR* strain showed significantly reduced virulence compared to strains with intact *hmuR* (hazard ratio, 0.128; 95% CI = 0.032 to 0.502; *P* = 0.003, with strain D3 as a reference). These results indicated that an intact Hmu system played an important role in bloodstream infections.

## DISCUSSION

To survive in iron-limited conditions, K. pneumoniae strains have evolved multiple iron uptake systems, which can be mainly summarized into three categories (26). The most important category is siderophores, such as salmochelin (IroBCDN), yersiniabactin (Irp1, Irp2, YbtAEPQSTUX, and FyuA), and aerobactin (IucABCDIutA), which are associated with enhanced virulence (25). The second category is iron ion uptake systems, including ferrous ion (FeoAB and SitABCD) and ferric ion (KfuABC) uptake systems (7). The third category is the HmuRSTUV heme/hemin uptake system. In the present study, we established a novel typing scheme for K. pneumoniae based on *hmuRSTUV* allelic profiles. Siderophore salmochelin is encoded by the *iro* locus (*iroBCDN*), aerobactin is encoded by the *iuc* locus (*iucABCDiutA*), and yersinibactin is encoded by the *ybt* locus (*irp1*, *irp2*, *ybtAEPQSTUX*, and *fyuA*). Lam et al. systematically analyzed the genetic diversity of these three siderophores and accordingly established novel typing schemes, namely, SmST, AbST, and YbST (24, 25). *iro* and *iuc* were detected at low prevalence (<10%), and the *ybt* locus was detected in 40% of the K. pneumoniae genomes (24, 25). These three siderophores were frequently associated with an elevated virulence of K. pneumoniae. In contrast, *hmu* was detect in most of K. pneumoniae genomes (98.9%) in this study, suggesting that the *hmu* locus was conserved in the genome of K. pneumoniae. Among 2,503 K. pneumoniae genomes, 62 AbSTs and 35 SmSTs were identified (25). Partly due to the relative higher prevalence and longer sequence of the *ybt* locus, 329 YbSTs were identified from 2,498 K. pneumoniae genomes (24). In our study, the *hmu* locus was much more prevalent, and 446 hmSTs were identified from 2,242 genomes, indicating that the hmST typing scheme might provide a novel strategy for monitoring and researching the molecular epidemiological characteristics of K. pneumoniae, regardless of lineage, virulence, or antimicrobial resistance.

Besides via degradation of erythrocyte in circulation, host heme could be acquired from diet absorption. Heme is one of the predominant forms of iron in the human diet (17), especially in meat, poultry, and fish (27). Consequently, heme transporters have been identified in human intestinal epithelial cells (28). Therefore, it is important to understand how Hmu influences the fitness and survival advantages of K. pneumoniae strains in the gut and further causes colonization or infections. Interestingly, hmSTs are more diverse in hypervirulent lineages. In our previous study, we found that the average difference of core genomic profile in hvKP strains was significantly larger than that in MDR strains (23), indicating greater genome diversification among hvKP strains. This is coincident with our finding that hmST was more diversely distributed among hvKP strains than among MDR strains. The genetic backgrounds—including chromosomal recombination, surface polysaccharide locus diversity, pangenome, plasmid, and phage dynamics—were different between these two lineages (5). In this study, hmST1 was only observed in hypervirulent ST23 strains, while hmST8 only existed in ST11, ST258, and ST512 MDR strains, all of which belong to CG258. Moreover, hypervirulent and MDR lineages had no shared hmSTs, suggesting that the *hmu* locus might be lineage specific. Similarly, other siderophore loci typing methods (such as AbST based on *iuc* loci and SmST based on *iro* loci) also revealed that hypervirulent and MDR lineages were associated with different allelic profiles. For example, hvKP lineages frequently harbored *iuc1/iro1* and *iuc2/iro2*, whereas *iuc5/iro5* were usually associated with MDR lineage CG258 (25). Also, in our previous study on the genetic diversity of siderophore loci, MDR lineage ST11 strains were associated with the yersiniabactin locus *ybt9*, while hypervirulent ST23 was associated with *ybt1* (29). Compared to *ybt*, *iro*, and *iuc*, the *hmu* locus was significantly more common in K. pneumoniae and showed greater allelic diversity, which made hmST suitable for almost all strains of the K. pneumoniae species complex. To summarize, these evidences suggested that the hmST scheme was useful in typing and distinguishing hvKP from MDR *K. pneumoniae*.

For the use of hmST, all *hmuRSTUV* allelic sequences could be downloaded from the Ridom website (https://www.cgmlst.org/ncs/schema/). The software Ridom SeqSphere+ could be used to acquire the *hmuRSTUV* alleles of a new genome. To identify the hmST for a new genome, one can directly acquire the allelic profiles of *hmuR*, *hmuS*, *hmuT*, *hmuU*, and *hmuV* by Ridom SeqSphere+ or Blast. Each unique combination of alleles was assigned an hmST. By searching Table S1, one can get the hmST of the new genome. In the future, the development of a more user-friendly tool for hmST (such as a web-interactive tool) is needed.

As shown in the growth curves, the growth effect of the *hmuR* mutation is more significant in strain D3 than in strain ZKP51; this might also have resulted from a genetic difference between the hvKP and MDR lineages. D3 is a hypervirulent strain producing four types of siderophores, i.e., enterobactin, salmochelin, yersiniabactin, and aerobactin, whereas ZKP51 is a CRKP strain that produced only enterobactin. We can therefore hypothesize that D3 required a much greater demand for iron. Thus, with hemin as the only source of iron, the inactivation of *hmuR* might have much more impact on the growth in D3 than the growth of ZKP51. The growth discrepancy between *hmuR* mutants and wild-type strains became larger as the hemin supplement increased from 1.25 to 2.5 μg/mL, suggesting the importance of an intact Hmu system in the presence of hemin. Therefore, in the bloodstream, where free heme could be released from hemoglobin, an intact Hmu system might facilitate bacterial growth and cause worse clinical outcomes in bloodstream infections.

In conclusion, HmuRSTUV is a critical iron uptake system in K. pneumoniae strains. The encoding locus *hmuRSTUV* was conserved in K. pneumoniae genomes, but genetic diversity was observed in *hmuRSTUV* allelic profiles. Based on the allelic profiles of *hmuRSTUV*, a novel typing scheme, hmST, was established. hmSTs were more diversely distributed in hypervirulent lineages than in MDR lineages. Interestingly, hypervirulent and MDR lineages had no shared hmSTs, suggesting that the typing scheme might be useful in distinguishing hvKP from MDR *K. pneumoniae*. The Hmu system played an important role in bloodstream infections, and hmST might provide an alternative strategy for monitoring and researching the molecular epidemiological characteristics of K. pneumoniae.

## MATERIALS AND METHODS

### Bacterial genomes and strains.

The study genomes constituted of 1,970 genomes from Pathogenwatch (https://pathogen.watch/) and 272 genomes from our previous study, which covered 20 tertiary hospitals in China (23). All the 1970 genomes from Pathogenwatch (up to 20 April 2020) were downloaded as study genomes without selection. All of the information concerning these genomes (including isolating times, locations, infection types, and accession numbers) are listed in Table S2.

Isolates D3 and ZKP51 were selected to determine the function of HmuR. D3 and ZKP51 were representatives of the hypervirulent and MDR lineages, respectively. D3 was an ST23 and K1 strain, isolated from clinical liver abscess. D3 produced four types of siderophores, i.e., enterobactin, salmochelin, yersiniabactin, and aerobactin. ZKP51 was a K. pneumoniae strain isolated from a patient with bloodstream infection. ZKP51 belonged to ST11 and produced only enterobactin.

### Typing scheme based on *hmuRSTUV* locus.

The publicly known hypervirulent K. pneumoniae genome NTUH-K2044 (accession no. NC_012731.1) served as the reference. The allelic numbers of *hmuR* (KP1_RS20345), *hmuS* (KP1_RS20350), *hmuT* (KP1_RS20355), *hmuU* (KP1_RS20360), and *hmuV* (KP1_RS20365) for NTUH-K2044 are designated as “1”; other *hmuRSTUV* alleles are referred to the scheme on Ridom SeqSphere+ website (https://www.cgmlst.org/ncs/schema/). Up to 3 February 2021, the numbers of alleles for *hmuR*, *hmuS*, *hmuT*, *hmuU*, and *hmuV* were 1,077, 794, 683, 639, and 606, respectively. Using Ridom SeqSphere+ software (v5.1.0), all of the collected genomes were mapped with the known alleles, so as to acquire the combinations of the *hmuRSTUV* alleles. Like MLST by seven housekeeping genes, the allelic profiles of *hmuRSTUV* were employed for typing K. pneumoniae, namely, hmST. The accumulation curve analysis and tanglegram (in which two rooted phylogenetic trees were drawn opposite each other) were performed using vegan and ggtree package, respectively, in R software.

### Knocking out and *in situ* complementation.

To determine the function of Hmu, mutagenesis and complementation of *hmuR* were performed via a CRISPR-Cas9-mediated genome-editing method (30). Generally, the single-guide RNA (sgRNA) directs the Cas9 protein to a target sequence in the presence of a 5′-NGG-3′ protospacer adjacent motif (PAM). Cas9 nuclease then cleaves the target sequence to cause a double-strand break. To repair the break, homologous recombination occurs in the presence of exogenously supplied donor DNA repair templates.

Specifically, a 20-bp spacer sequence before a PAM site (5′-NGG-3′) in the target sequence was ligated into plasmid pSGKP between two reversed *BsaI* sites, which was located between the J23119 promoter and the sgRNA scaffold (30). For *hmuR* knockout, the donor DNA was generated by connecting the forward and reverse homology arms by overlap PCR. Cotransformation of the spacer introduced the pSGKP plasmid and the donor DNA into the l-arabinose-induced recipient cells harboring the pCasKP plasmid, which expressed the Cas9 protein and lambda Red recombination system. The LB agar plate containing 50 μg/mL apramycin and 100 μg/mL hygromycin was used to screen the correct transformants at 30°C overnight. The successful knocking out was verified by PCR and Sanger sequencing. The process of *in situ* complementation was the same as for the knockouts. Of note, a silent mutation should be introduced in the PAM site on donor DNA to prevent the cutting of Cas9 when performing complementation. The primers used in this study are listed in Table 1, and the fragment for *hmuR* knockout and complementation is indicated in Fig. 1B. A detailed protocol on performing the knockout and *in situ* complementation is included in the supplemental material.

**TABLE 1:** Oligonucleotides used in the study

Primer	Sequence (5′–3′)^*a*^
*hmuR*-KO-donor-F1	TCCGTCAGCAGCATGGTGTA
*hmuR*-KO-donor-R1	GAGCCGAAATCGTTAGCGCC
*hmuR*-KO-donor-F2	GGCGCTAACGATTTCGGCTCCGCGAAAGCTACAGCAACAG
*hmuR*-KO-donor-R2	AGTCGACGTTGACGCCC
*hmuR*-KO-spacer-F	TAGTCCATGACTGGACTTATATGC
*hmuR*-KO-spacer-R	AAACGCATATAAGTCCAGTCATGG
*hmuR*-comp-donar-F	CGGTGGATGACACCTCGGAACAAATGACCGTCACCGCCCCCGCGCCGGTGCAGAAAGCCGGTAGCGAACACAGCATCAGCGCCCGAGAG
*hmuR*-comp-donar-R	GCTTTATCGTTGGCGTCCTG
*hmuR*-comp-spacer-F	TAGTGCGAACACAGCATCAGCGCC
*hmuR*-comp-spacer-R	AAACGGCGCTGATGCTGTGTTCGC
*hmuR*-F	GTATGGAGAGCAACCGCGTA
*hmuR*-R	GATGTTCGACGGCACATTCG
pCasKP-conf-F	GGGAAATACAGACCGCCACA
pCasKP-conf-R	TGAAGCTGATAGGGGAGCCT
pSGKP-conf-F	AGGATTTGCAGACTACGGGC
pSGKP-conf-R	TGTGTGGAATTGTGAGCGGA

a BsaI sites are indicated by underlining.

Plasmid pCasKP contained the temperature-sensitive replicon *repA101*(Ts) and pSGKP harbored the sucrose-sensitive gene *sacB* (30). To cure these two plasmids after successful editing, cells were streaked onto a Luria-Bertani (LB) agar plate containing 5% sucrose and incubated at 37°C overnight. Several colonies were plated onto LB plates with or without apramycin or hygromycin supplementation, respectively. The colonies with the successful curing of both plasmids could only grow on the plate without antibiotic.

### Growth curve.

The growth curve analysis was performed as previously described (31). Briefly, three independent cultures for each strain were grown overnight in Mueller-Hinton (MH) broth and diluted to 1:1,000 in M9 standard medium with bovine hemin supplement. To ensure that that only hemin could be used as an iron source, 250 μM 2,2′-bipyridyl (BIP) was added to M9 medium. Three replicates of each culture (200 μL) were added to a flat-bottom 100-well plate. The plate was incubated at 37°C with agitation, and the optical density at 600 nm (OD_600_) values of each culture were recorded every 5 min by using a Bioscreen C automated microbiology growth curve analysis system (Oy Growth Curves Ab, Ltd., Turku, Finland). OD_600_ values of media without bacteria were used to normalize the initial OD for each culture.

### Mouse lethality assay.

D3, D3Δ*hmuR*, and D3Δ*hmuR*::*hmuR* strains were used to estimate the role of HmuR in bloodstream infection. We added 20 μL of overnight-cultured strain into 2 mL of MH broth, followed by incubation for 3 h until the samples reached the mid-log phase. All cultures were centrifuged at 6,000 rpm for 5 min, and the supernatants were discarded. We washed the cells with phosphate-buffered saline once and then resuspended them. Cell suspensions of 3 × 10^6^ CFU (0.2 mL) were injected into the caudal veins of 6- to 8-week-old BALB/c mice. Ten mice were used as a sample population for each strain. The mortality rates were recorded for 5 days postinjection. All procedures performed in studies involving animals were approved by the Institutional Animal Care and Use Committee of Sir Run Run Shaw Hospital.

### Data availability.

All the sequence accession numbers of the study genomes were available in the supplemental materials.
