# Supplementary material for: Development of a Novel Typing Scheme Based on the Genetic Diversity of Heme/Hemin Uptake System Hmu in Klebsiella pneumoniae Species Complex
Source: Microbiol Spectr. 2023 Feb 14;11(2):e01062-22. doi: 10.1128/spectrum.01062-22 (PMC10101058; doi:10.1128/spectrum.01062-22)
Supplement: Supplemental file 3 — Supplemental material. Download spectrum.01062-22-s0001.pdf, PDF file, 0.9 MB [file spectrum.01062-22-s0001.pdf]

Online Supplemental Data

I. Supplemental material for results

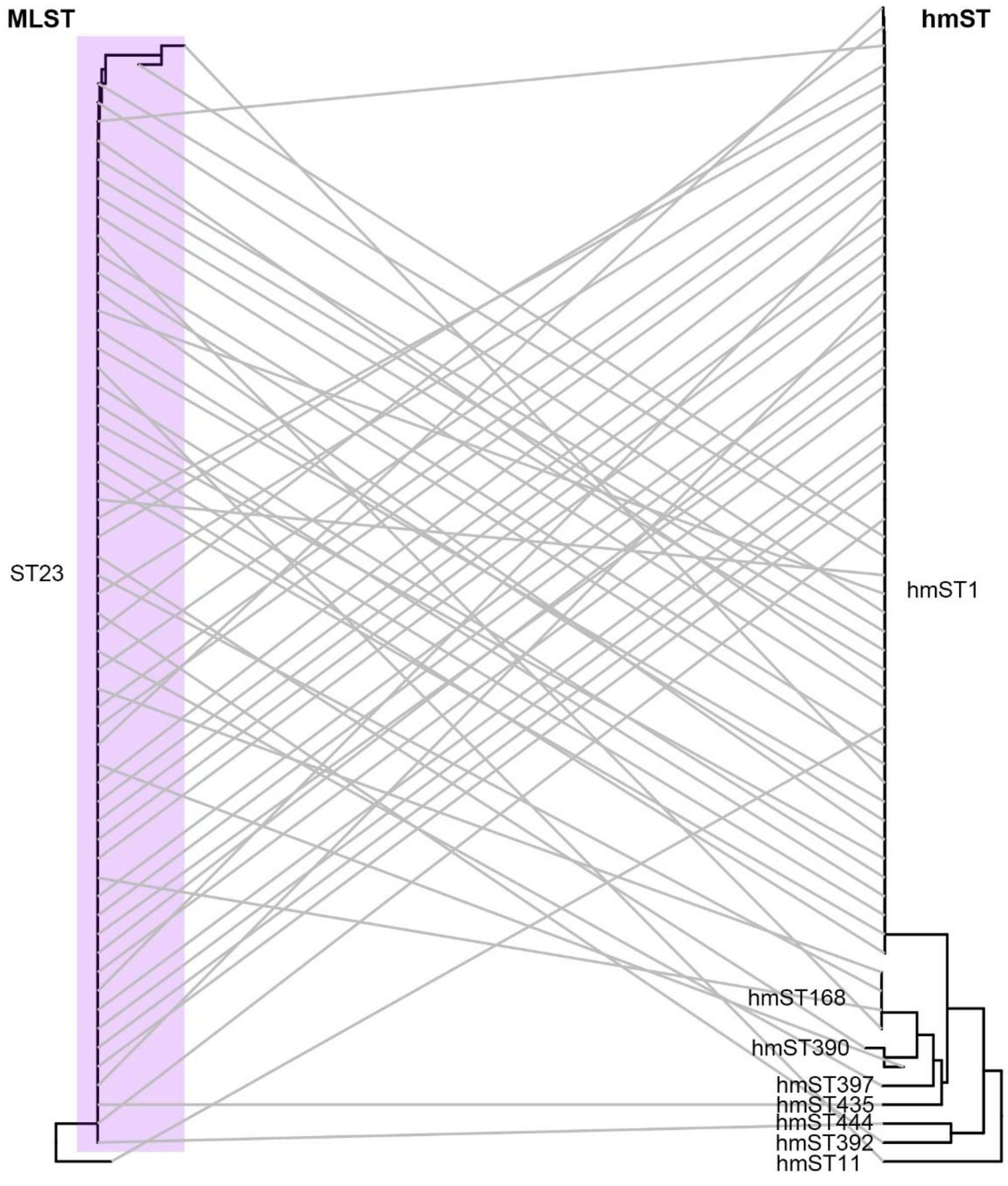

Figure S1 Subset of phylogenies of ST23 hypervirulent strains inferred from MLST (left) and hmST (right). Matching strains were connected using grey auxiliary lines.

II. The detailed procedures for KO and *in-situ* complementation in this study

Part A．*hmuR* KO

1. Linearize sgRNA expression plasmids pSGKP with *Bsa*I：

pSGKP plasmid	1 μg
10 × Cutsmart buffer (NEB)	5 μL
*Bsa*I-HF (NEB)	1 μL
ddH_2O	Add to 50 μL

Incubate the solution at 37 °C for 1 hour. Purify the digested plasmid and quantify the DNA concentration using Nanodrop. The purified plasmid can be stored at -20℃ and also used in *hmuR* complementation.

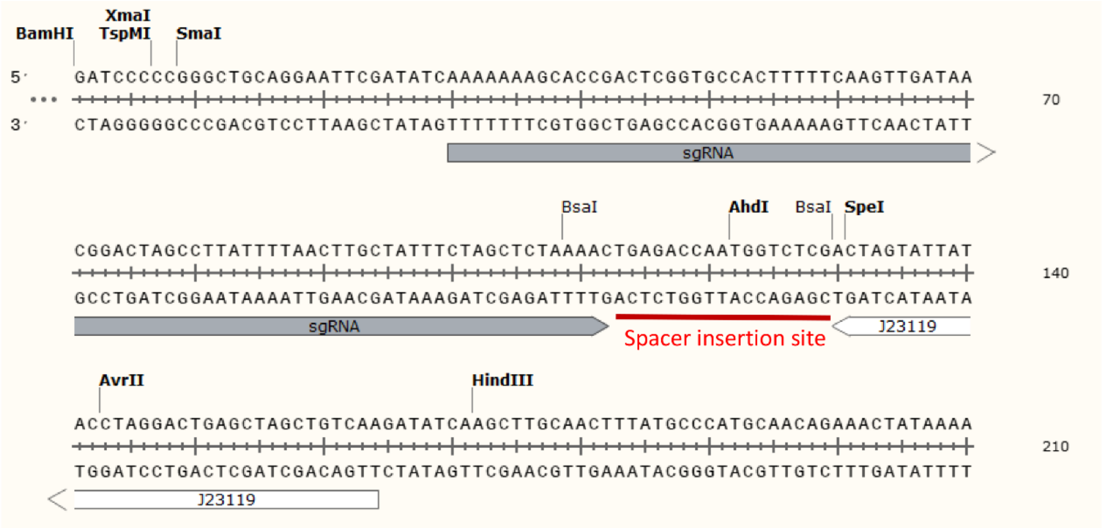

Figure S2 Sequence of the sgRNA expression cassette. The expression of sgRNA is driven by the synthetic strong constitutive J23119 promoter. The two BsaI sites are used for seamless and one-step assembly of spacers.

2. Spacer design:

Select a 20 bp-spacer sequence before a PAM site (5'-NGG-3') in the *hmuR* sequence. Design and synthesize two oligos in the following forms:

hmuR-KO-spacer-F: 5'- TAGTCCATGACTGGACTTATATGC-3'

hmuR-KO-spacer-R: 5'- AAACGCATATAAGTCCAGTCATGG-3'

3. Phosphorylation and annealing of the spacers:

Spacer-F (100 μM)	5 μL
Spacer-R (100 μM)	5 μL

10 × T4 DNA Ligase buffer (NEB)	5 μL
T4 Polynucleotide Kinase (NEB)	1 μL
ddH_2O	34 μL

Phosphorylate the spacers at 37 ℃ for 30 min. Incubate the reaction solution at 95 ℃ for 3 min and then slowly cool it down to room temperature. Dilute the annealed spacers 100 times using ddH_2O.

4. Ligate spacers into the linearized sgRNA expression plasmid

Linearized sgRNA plasmid	20-50 ng
Diluted annealed spacers (1:100 dilution)	1 μL
10 × T4 DNA Ligase buffer (NEB)	1 μL
T4 DNA Ligase (NEB)	0.5 μL
ddH_2O	Add to 10 μL

Incubate at room temperature overnight.

5. Transformation

10 μL ligated product was transformed into 100 μL *E. coli* DH5α competent cells and incubated at 37 ℃ overnight on plates containing 100 μg/mL hygromycin. The successful cloning of the spacer was verified by PCR with the primers of pSGKP-conf-F/R and by sequencing. The transformants can be stored at -80 ℃.

6. Transforming pCasKP into D3 and ZKP51

10 μL pCasKP plasmid was transformed into 100 μL D3 and ZKP51 competent cells and incubated at 30 ℃ overnight on plates containing 50 μg/mL apramycin. The successful cloning of the spacer was verified by PCR with the primers of pCasKP-conf-F/R and by sequencing. The transformants can be stored at -80 ℃.

7. Donar template design:

The donor template for repair of the dsb was generated via overlap PCR. The forward homology arm was generated by PCR with *hmuR*-KO-donar-F1/R1 while the reverse homology arm was generated by PCR with *hmuR*-KO-donar-F2/R2. Since the end of the PCR product #1 and the start of the PCR product #2 shared a 20-bp sequence, the two sequences could be connected via a third PCR procedure with *hmuR*-KO-donar-F1/R2 (Figure S2). Purify the donar template and store at -20°C.

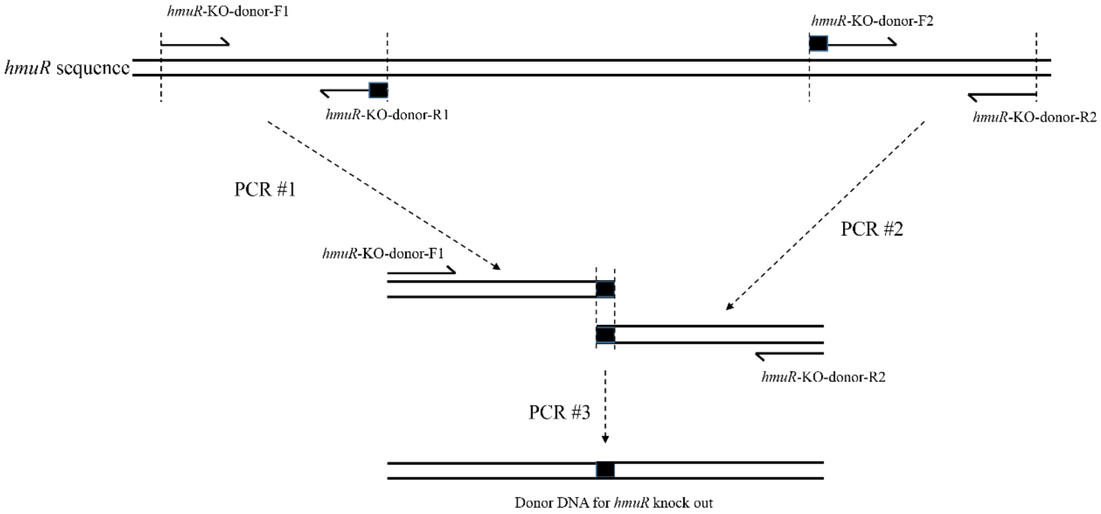

Figure S3 The outline of the overlap PCR in this study

8. *hmuR* KO

Co-transform the spacer-introduced pSGKP plasmid and the donor DNA into the L-arabinose-induced recipient cells harboring the pCasKP plasmid by electroporation. The cells were plated onto a LB agar plate containing 50 μg/mL apramycin and 100 μg/mL hygromycin. The plate was incubated at 30 ℃ overnight. The successful editing was verified by PCR with the primers of *hmuR*-KO-donar-F1/R2 and sequencing.

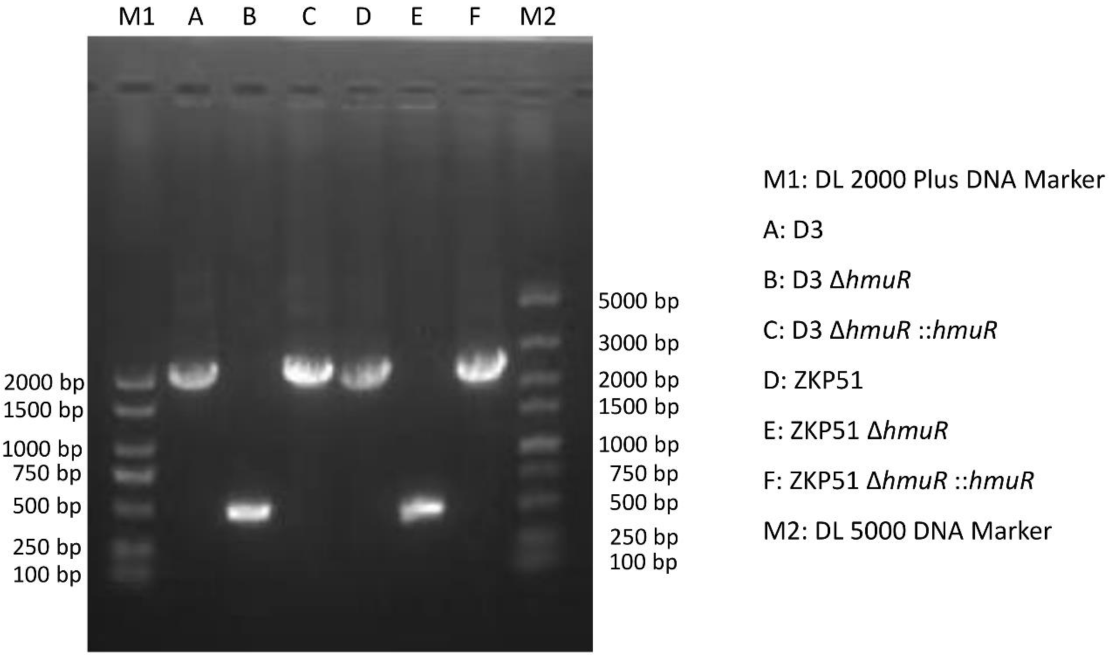

Figure S4 The successful KO and complementation were verified by PCR with the primers of hmuR-KO-donar-F1/R2 and sequencing. The gel electrophoretic pattern indicated the length of PCR product for WT and complemented strains were 2345 bp,

while the length of truncated hmuR was 499 bp.

Part B．*hmuR in-situ* complementation

1. Linearize sgRNA expression plasmids pSGKP with *Bsa*I (same as Step 1 in Part A)

2. Spacer design:

Select a 20 bp-spacer sequence before a PAM site (5’-NGG-3’) in the truncated *hmuR* sequence. Design and synthesize two oligos in the following forms:

hmuR-comp-spacer-F: 5’- TAGTGCGAACACAGCATCAGCGCC-3’

hmuR-comp-spacer-R: 5’- AAACGGCGCTGATGCTGTGTTCGC-3’

3. Phosphorylation and annealing of the spacers (same as Step 3 in Part A)

4. Ligate spacers into the linearized sgRNA expression plasmid (same as Step 4 in Part A)

5. Transformation (same as Step 5 in Part A)

6. Transforming pCasKP into *hmuR* mutants of D3 and ZKP51 (same as Step 6 in Part A)

7. Donar template design:

To prevent the cutting of Cas9/sgRNA complex in the genome of edited cells, a silent mutation was introduced in the PAM. To achieve this, we prolonged the *hmuR*-comp-donar-F and a silent mutation was introduced (CGG→CGA). The donor template for complementation was then generated with *hmuR*-comp-donar-F/R. Purify the donar template and store at -20℃.

8. *hmuR in-situ* complementation

Co-transform the spacer-introduced pSGKP plasmid and the donor DNA into the L-arabinose-induced recipient cells harboring the pCasKP plasmid by electroporation. The cells were plated onto a LB agar plate containing 50 μg/mL apramycin and 100 μg/mL hygromycin. The plate was incubated at 30 ℃ overnight. The successful editing was verified by PCR with the primers of *hmuR*-F/R and sequencing. The cells with successful *in-situ* complementation possess an intact *hmuR* sequence but with a silent mutation at 69th amino acid (CGG→CGA, Arginine).

Part C. Plasmid curing

Both pSGKP and pCasKP were acquired plasmids, which need to be cured after gene editing. Inoculate a colony containing the desired mutation into antibiotic-free LB broth medium and incubate it at 37 °C for 12 h. Then, a fraction of the culture was streaked onto a LB agar plate

containing 5% sucrose. The plate was incubated at 37 °C for 12 h. Several colonies were streaked onto LB plates with or without the supplementation of apramycin or hygromycin, respectively, to confirm the successful curing. The colonies with the successful removal of both the pCasKP and pSGKP plasmids could only grow on the plate without any antibiotics.
